# Women’s health in focus: Real-world data on valproate prescriptions during pregnancy – a cohort study in Catalonia (Spain)

**DOI:** 10.1136/bmjopen-2024-085167

**Published:** 2024-08-12

**Authors:** Lucía Bellas, Lina Camacho-Arteaga, Maria Giner-Soriano, Albert Prats-Uribe, Cristina Aguilera, Cristina Vedia Urgell, Antonia Agustí

**Affiliations:** 1 Department of Clinical Pharmacology, Hospital Universitari Vall d'Hebron, Barcelona, Spain; 2 Department of Pharmacology, Therapeutics and Toxicology, Universitat Autonòma de Barcelona, Barcelona, Spain; 3 Medicines Research Unit, Institut Universitari d'Investigació en Atenció Primària Jordi Gol (IDIAP Jordi Gol), Barcelona, Spain; 4 Universitat Autònoma de Barcelona, Bellaterra (Cerdanyola del Vallès), Spain; 5 Nuffield Department of Orthopaedics Rheumatology and Musculoskeletal Science, University of Oxford, Oxford, UK; 6 Unitat de farmàcia. Servei d’Atenció Primària Barcelonès Nord i Maresme, Institut Català de la Salut, Badalona, Spain; 7 Departament de Farmacologia i Terapèutica, Universitat Autònoma de Barcelona, Bellaterra, Cerdanyola del Vallès, Spain

**Keywords:** pregnant women, electronic health records, maternal medicine, clinical pharmacology, public health

## Abstract

**Abstract:**

**Objectives:**

To characterise the exposure to valproate within a cohort of pregnant women using electronic health records (EHRs) from Catalonia (System for the Development of Research in Primary Care, SIDIAP).

**Design:**

Drug-utilisation cohort study covering the period from January 2011 to June 2020. The study included pregnancy episodes of women from Catalonia identified by the algorithm.

**Setting:**

Data were sourced from SIDIAP, a comprehensive EHR repository that includes information from various data sources: recorded prescriptions (both hospital and primary care), diagnoses and sociodemographic characteristics identified by primary care physicians, and sexual and reproductive health data from ASSIR (used by gynaecologists and midwives).

**Participants:**

Women aged 12–50 with at least one pregnancy episode occurred during January 2011–June 2020 and at least a prescription of valproate during pregnancy.

**Primary and secondary outcomes:**

Primary outcomes included valproate exposure, measured through prevalence and cumulative incidence in pregnancy episodes and by trimester. The impact of regulatory measures (risk mitigation measures, RMMs) was assessed, and prescriptions over time were analysed using interrupted time series analysis. Secondary outcomes included health issues, pregnancy outcomes, smoking habits and socioeconomic characteristics.

**Results:**

A total of 99 605 pregnancies were identified, with at least 3.03‰ (95% CI 2.69‰ to 3.39‰) exposed to valproate at some point (302 pregnancies, 276 women). The median pregnancy duration was 38.30 weeks (IQR 12.6–40.1), and the median age at pregnancy was 32.37 years (IQR 27.20–36.56). Epilepsy was the most frequent health issue. The prevalence and cumulative incidence of valproate prescriptions decreased during pregnancy and increased postpregnancy. The RMMs implemented in 2014 led to a reduction in monthly valproate prescriptions during pregnancy in this cohort.

**Conclusions:**

The study highlights the decline in valproate prescriptions during pregnancy due to RMMs and underscores the need for standardised methodologies in future studies to ensure the safety of pregnant patients and optimise scientific evidence.

**STRENGTHS AND LIMITATIONS OF THIS STUDY**

- The study benefits from a validated methodology, including the use of a validated database, ensuring the reliability of the data.
- The validated database encompasses information from prescriptions uploaded to e-CAP system (outpatient prescriptions from both primary care and hospitals), sociodemographic data from primary care, and sexual and reproductive health modules (ASSIR).
- Data are extracted from electronic health records; therefore, these data are not specifically tailored for research purposes.
- We used prescription data to assess antiseizure medication exposure, which means that billing data and adherence to medication were not considered.
- The present study lacks some clinical aspects of pregnancies that are followed up in hospitals (referred from primary care because of complications) or in private settings.

## Introduction

 Valproate and its related compounds are approved in Spain for the treatment of epilepsy, encompassing primary generalised epilepsy, partial epilepsy, secondary generalised seizures and West and Lennox-Gastaut syndrome. Additionally, it holds approval for the treatment of acute mania in select cases among individuals with bipolar disorder.^1^,^3^ The teratogenic risk associated with the use of valproate in pregnant women is well established.^4^,^7^ Therefore, its use in women of childbearing age is restricted to prevent valproate exposure during conception and pregnancy. The best-known malformations are neural tube defects, but valproate has been shown to cause neurodevelopmental changes and other congenital malformations.^8 9^

There have been several warnings regarding the use of valproate in women of childbearing age: In October 2014, the European Medicines Agency (EMA) Pharmacovigilance Risk Assessment Committee (PRAC) issued recommendations for valproate prescribing. These recommendations emphasised that valproate and related substances should not be used in girls, women of childbearing potential and pregnant women unless alternative treatments are ineffective or not tolerated. Specifically, they should be contraindicated in prophylaxis of migraine attacks in pregnancy and women of childbearing potential who are not using effective methods of contraception.^10^ In March 2018, the PRAC recommended update risk mitigation measures (RMMs), introducing a Pregnancy Prevention Programme (PPP) and advocating for more stringent measures.^11^

There are several studies which have explored risk awareness, prescription patterns^12^,^14^ and adherence to the RMMs in different European countries. As an example, the multidatabase longitudinal study carried out by Abtahi *et al*,^15^ spanning from January 2010 to December 2020, assessed the impact of prevention programmes for valproate-containing drugs. Despite a reduction in valproate prevalence, there was no statistically significant reduction in incident use in women of childbearing age. Poor adherence to contraception during valproate treatment and high rates of concomitant pregnancy were observed. Implemented measures had limited impact, as indicated by data from five European databases. In the same field of study, Jödicke *et al*^16^ reported on valproate use in women aged 12–55 in the EU (European Union), analysing data from six electronic healthcare databases. Both studies suggest a decline in valproate use among women of childbearing age in most European databases, with an increase in alternative treatments, such as lamotrigine and levetiracetam among other drugs.

The findings mentioned above, along with other studies,^17^,^19^ highlight the significance of regulatory measures and electronic healthcare databases in assessing prescription patterns during pregnancy.^20^ In this context, Lestón *et al* developed an algorithm within the System for the Development of Research in Primary Care (SIDIAP) database to identify pregnancy episodes from January 2011 to June 2020, which seems to hold the potential as an efficient database for investigating drug safety during pregnancy and its implications for the offspring.^21^ In this context, assessing valproate prescription patterns during pregnancy has considerable value, due to the lack of drug-safety trials in this population, the well-documented impact on the offspring and the institutional efforts to prevent prescriptions in pregnant women.

### Objective

The present study sought to characterise the prevalence and cumulative incidence of valproate prescriptions, along with alternative prescriptions, within a cohort of pregnant women using an electronic health records (EHR) database from Catalonia (SIDIAP).

## Design and methods

### Study

An observational cohort drug-utilisation study, spanning from January 2011 to June 2020.

### Data source

Our dataset originates from the SIDIAP,^22 23^ a comprehensive repository that captures clinical data of around 5.8 millions of people living in Catalonia, constituting around 80% of the regional population. This information is pseudonymised and stems from various data sources, primarily the EHR of the Catalan Health Institute. The EHR includes a wealth of information, encompassing sociodemographic characteristics, health issues recorded using International Classification of Diseases (ICD)-10 codes^24^ and details on toxic habits. This particular information is recorded by the primary care physician during women’s visits. We also assessed drug prescriptions issued in primary healthcare (PHC), categorised under the Anatomical Therapeutic Chemical (ATC) classification system. Both hospital and primary care prescriptions are consolidated within the same system (e-CAP system).^25^ Additionally, our database incorporates records from the sexual and reproductive healthcare module (ASSIR: Sexual and reproductive healthcare records), offering a comprehensive overview of pregnancies. This module captures crucial data such as the date of the last menstrual period (LMP), gestational week and details on delivery or pregnancy termination outcomes.

### Pregnancies and women

The algorithm developed to detect pregnancy episodes in the SIDIAP database, fully described elsewhere,^21^ identified episodes based on variables like the first day of the LMP, reasons for pregnancy termination and diagnoses recorded in PHC records. Multiple pregnancy episodes could be recorded for each pregnant woman. We only considered full pregnancy episodes (those whose date of onset and date of ending were included in the whole observation period).

### Pregnancy outcomes

We evaluated the diagnostic outcome associated with all exposed episodes. These diagnoses could include vaginal delivery, abortion (including induced abortion and miscarriage), caesarean section, prematurity, fetal death, ectopic pregnancy or hydatiform molar.

### Health issues, smoking habits and socioeconomic characteristics of pregnancy episodes

We explored diverse characteristics of exposed pregnancy episodes (at least one active prescription during that pregnancy episode) to valproate. Health issues correspond to health problems identified through ICD-10 codes and recorded by the primary care physician during regular visits. In our case, these health issues are not associated with a particular indication but have been studied comprehensively for each patient, considering all the health problems that the woman had active during the year preceding the pregnancy onset date. The ICD-10 codes are shown in online supplemental table 1S. We excluded pregnancy-related diagnoses. Regarding smoking habit (classified as non-smoker, active smoker and history of smoking habit) and social class based on the MEDEA (Mortalidad en áreas pequeñas Españolas y Desigualdades socioEconómicas y Ambientales) index—a deprivation index based on five indicators related to work, education, housing conditions calculated at the census tract level and available for urban areas,^22 26^ our criteria also involved selecting those present 1 year prior to the pregnancy onset date as well.

### Exposure to valproate

Pregnancy episodes were systematically classified into trimesters (first, second and third trimester). We also evaluated exposure before and after pregnancy, comprising a preceding 3-month observation period and two subsequent phases of 3 and 6 months after the conclusion of the pregnancy (hereafter referred to as ‘pregnancy intervals’). Only those periods having a complete observation span between January 2011 and June 2020 were for analysis. Within the above-mentioned time frame, the prevalence and cumulative incidence of valproate prescriptions were assessed by trimester. For prevalent (or current) users, pregnancy intervals were considered as exposed if they overlapped with a prescription by at least 1 day. Incident (or new) users were identified if they initiated a prescription during that pregnancy interval since the study’s inception in January 2011, incorporating a 1-year washout period (period of time necessary without a prescription to consider a case as new) for incidence calculations from the day of pregnancy onset. Prescription duration and dose were not considered.

### Prescriptions over time

- PRAC intervention: We assessed the prevalence of prescriptions of valproate throughout the entire study observation period. Given the exclusive inclusion of complete pregnancy episodes, data scarcity was observed at the study’s commencement and conclusion. Consequently, we concentrated our evaluation on the prevalence from January 2012 to January 2020 to ensure a more thorough analysis. We opted to examine the impact of the 2014 measures over those implemented in 2018 due to the scarcity of data within the limited time frame from March 2018 to January 2020. Plus, these measures were launched in March 2018 but only implemented in Spain in December 2018.^14^ To assess the impact of the 2014 Risk Management Measures (RMMs) on the prevalence of valproate prescription during pregnancy, interrupted time series (ITS) analyses were conducted monthly. The impact of the measures was calculated using the beta coefficients of the ITS analyses, in the form of mean rate difference postintervention, p value and percentage of change compared with the mean counterfactual value had the intervention not occurred.
- Prevalence of ASM (antiseizure medication) over time: We assessed the monthly prevalence of prescriptions over time of the ATC drug groups, namely lamotrigine, levetiracetam and valproate, throughout the entire study observation period. We concentrated our evaluation on the prevalence from January 2012 to January 2020 due to de absence of data at the ending and beginning of the study.

### Statistical analysis

- Baseline demographic, clinical characteristics of patients and pregnancies episodes were described as mean and SD or median and quartiles for continuous variables, and as percentages for categorical variables.
- Prevalence and incidence were calculated per 1000 pregnancies with 95% CIs. They were computed using our custom software developed in R (V.4.3.1) in conjunction with the Incidence and Prevalence package.^27 28^
- Employing Poisson regression and ITS analyses at 96 monthly intervals, we assessed changes in VPA prescription prevalence preintervention and postintervention (2011–2014 and 2014–2020). Following Faraway’s^29^ recommendation and considering our Akaike information criterion (AIC), favouring the Poisson model (AIC=463.81), we adopted it to calculate the expected prevalence. We assessed autocorrelation by the introduction of lag variables^30^

### Patient and public involvement

Patients and public were not involved in the design, recruitment or conduction of the present study.

## Results

### Pregnancies and women

The number of pregnancies was 99 605 (which corresponds to 79 564 women), with a median of 39 weeks (IQR 34.5–40.1).

### Exposure to valproate

Of 99 605 pregnancies at least 3.03‰ (95% CI 2.69‰ to 3.39‰) were exposed at some point in their pregnancy to valproate (302 pregnancies, 276 women). The median duration of exposed pregnancies was 38.30 weeks (IQR 12.6–40.1).

### Health issues, smoking habits and socioeconomic characteristics

The mean age of pregnant women exposed to valproate during pregnancy was 31.68 (±6.83), with a median of 32.37 (IQR 27.20–36.56). The most frequently identified health issues were epilepsy (79.13%, 95% CI 74.54% to 83.71%), anxiety (68.87%, 95% CI 63.64% to 74.99%) and nicotine dependence (37.08%, 95% CI 31.62% to 42.5%). Regarding the smoking habits of the exposed cohort, not many pregnancy episodes had a registry of smoking status. Those who had it corresponded mostly to nonsmokers (9.6%). These findings are represented in table 1.

**Table 1:** Sociodemographic characteristics of pregnancies exposed to valproate

	N, % (95% CI)
Health issues	
Epilepsy	239, 79.13%, (74.54, 83.71%)
Anxiety disorder	208, 68.87%, (63.64, 74.09%)
Nicotine dependance	112, 37.08%, (31.62%, 42.5%)
Obesity	71, 23.50%, (18.718, 28.28%)
Pruritus	70, 23.17%, (18.41, 27.92%)
Major depressive disorder	58, 19.20%, (14.75, 23.64%)
Chronic obstructive pulmonary disease	45, 14.90%, (10.88, 18.91%)
Smoking habit	
Non-smoker	29, 9.6%, (6.27, 12.92%)
Active smoker	20, 6.62%, (3.81, 9.42%)
History of smoking habit	8, 2.64%, (0.83, 4.46%)
Missing values	245, 81.12%
MEDEA (Mortalidad en áreas pequeñas Españolas y Desigualdades socioEconómicas y Ambientales) index —quintiles	
High socioeconomic status	25, 8.27%, (5.17, 11.38%)
Moderate socioeconomic status	32, 10.59%, (7.12, 14.06%)
Average socioeconomic status	51, 16.88%, (12.66, 21.11%)
Low socioeconomic status	46, 15.23%, (11.17, 19.28%)
Extreme socioeconomic deprivation	46, 15.23%, (11.17, 19.28%)
Missing values	102, 33.77%
Population	
Urban areas	232, 76.82%, (72.06, 81.58%)
Rural areas	70, 23.17%, (18.41, 27.93%)

Most of the exposed episodes belonged to the most deprived quintiles in the MEDEA socioeconomic index—U4 and U5 (46 cases, 15.23%). ICD-10 codes and associated diagnoses, as well as MEDEA index are registered in online supplemental tables 1S and 2S.

### Pregnancy outcomes

Among women exposed to valproate during pregnancy, There were 164 cases of vaginal deliveries, which represents a 54.43% of the exposed cases (95% CI 48.68% to 59.92%), 89 cases of abortions (including those classified as ‘abortions’ and those classified as ‘voluntary interruption’), representing 29.47% (95% CI 24.32% to 34.61%) and 49 cases of caesarean delivery (16.22%, 95% CI 12.06% to 20.38%). There were no reported cases of prematurity, fetal death, ectopic pregnancy or hydatiform mole.

### Prevalence

**Figure 1:**
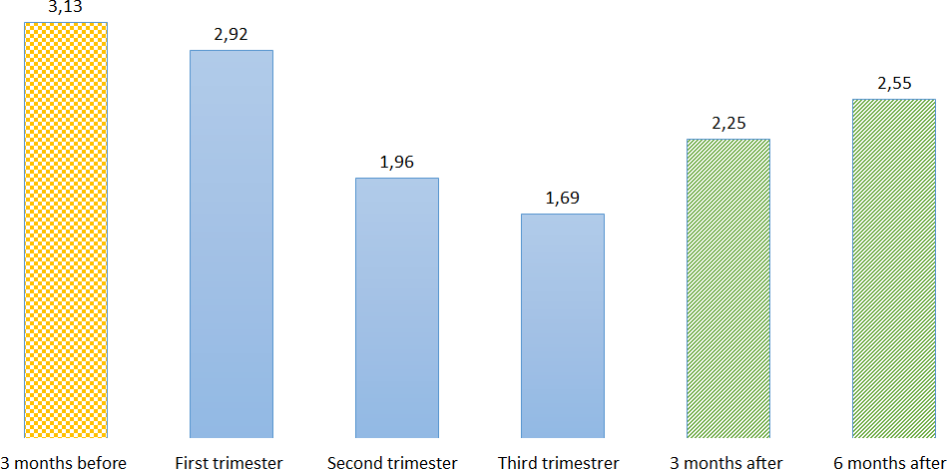
Prevalence of valproate prescriptions during pregnancy (%).

Prevalence of valproate use appears to decrease as pregnancy progresses, dropping from 3.13‰ (95% CI 2.80‰ to 3.51‰) in the 3 months before pregnancy interval and 2.92‰ in the first trimester (95% CI 2.60‰ to 3.28‰) to 1.96‰ in the second trimester (95% CI 1.76‰ to 2.29‰) and 1.69‰ in the third trimester (95% CI 1.42‰ to 2.00‰). However, in the 3 months following pregnancy, a spike in prevalence is observed, as it increases again to 2.25‰ (95% CI 1.96‰ to 2.57‰) (see figure 1, online supplemental table 3S).

### Incidence

**Figure 2:**
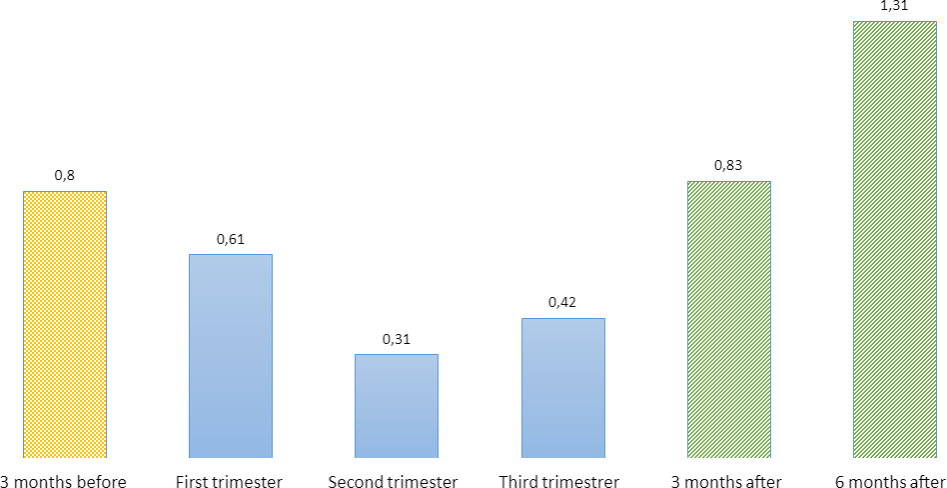
Cumulative incidence of valproate prescriptions during pregnancy (%).

We identified a total of 96 880 pregnancy intervals for the first trimester after applying a year of washout, of which 60 had an incident prescription of valproate. The cumulative incidence of valproate prescriptions decreases from 0.80‰ (95% CI 0.63‰ to 1.00‰) in the 3 months before pregnancy, continues to decline during the first (0.61‰, 95% CI 0.47‰ to 0.79‰) and second trimester (0.31‰, 95% CI 0.20‰ to 0.46‰) and experiences a spike in the third trimester (0.42‰, 95% CI 0.29‰ to 0.60‰), which corresponds to 8 cases. Furthermore, it increases even more in the pregnancy interval corresponding to the 3 months following the end of pregnancy (0.83‰, 95% CI 0.66‰ to 1.00‰) (figure 2, online supplemental table 4S).

### Prescriptions over time

**Figure 3:**
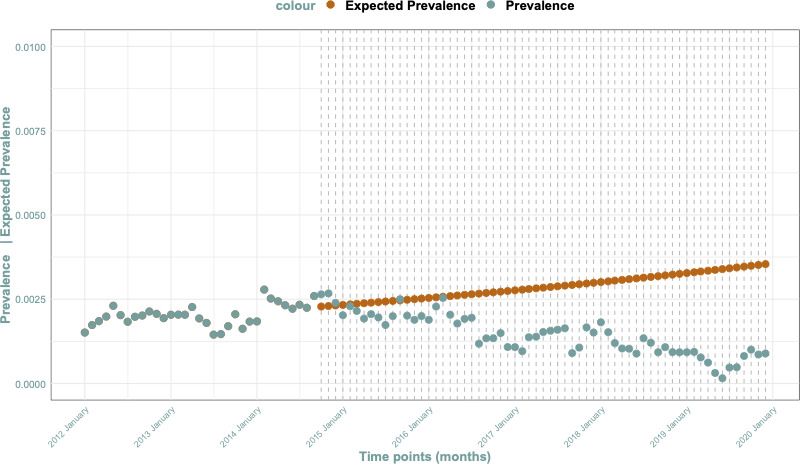
Impact of RMM. RMM, risk mitigation measure.

**Figure 4:**
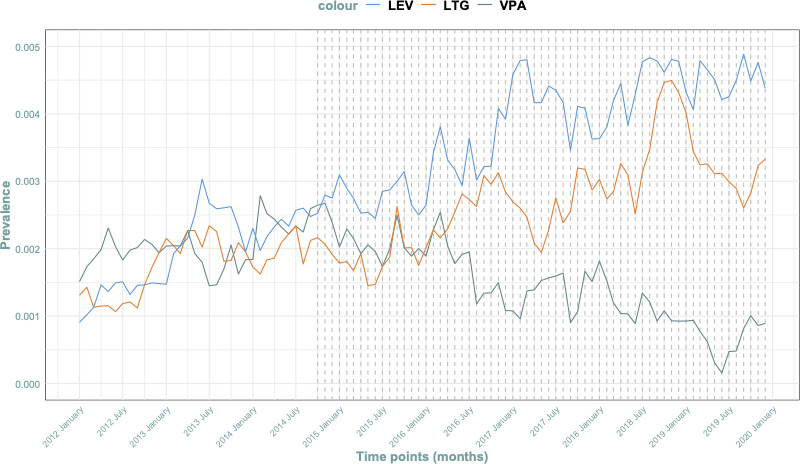
Prevalence of Valproate (VPA), levetiracetam (LEV) and lamotrigine (LGT) over time.

- PRAC intervention: Post-October 2014 measures led to a 2.73% (95% CI 1.78% to 3.68%) monthly decrease in valproate prescription cases during pregnancy. This finding is illustrated in figure 3. Monthly prevalence of valproate prescriptions is available in online supplemental table 5S.
- Prevalence of ASM over time: We observed a decrease in the monthly prescription prevalence of valproate, along with an increase in prescriptions for lamotrigine and levetiracetam, as shown in figure 4. Monthly prevalence of lamotrigine and levetiracetam prescriptions are available in online supplemental tables 6S and 7S. ATC codes and abbreviations can be found in online supplemental table 8S.

## Discussion

This study describes exposure to valproate during pregnancy, as well as the characteristics of the exposed pregnancies. We assessed the prescription of valproate in a cohort of pregnant women in Catalonia, dividing pregnancies by trimesters (pregnancy intervals). Of 99 605 identified pregnancies (in 76 459 women), at least 302 pregnancy episodes were exposed to valproate (276 women exposed in total). We also evaluated the impact of the RMMs recommended by the EMA.^10^

Our study shows a decrease in the prevalence and cumulative incidence of prescriptions of valproate as pregnancy progresses (prescriptions decline along pregnancy intervals). It is noteworthy that there is a subsequent increase in prescriptions after the ending of pregnancy, even though the drug is excreted in breast milk.^3^

Cumulative incidence by trimester has not been analysed in any other study on record. The notable rise in incident cases (eight cases) during pregnancies entering the third trimester is striking. This increase could be associated with the reintroduction of the drug in patients who have not responded well to alternative medications or even due to the escalation of epileptic seizures in the third trimester.^31^ Furthermore, there might be a misconception that the risk of malformations is primarily linked to exposure in the first trimester, overlooking the potential risk associated with developmental disorders.

Overall prevalence, prevalence by trimesters and prescription patterns, align with findings from research carried out in other countries,^14 20^ especially those corresponding to Italian databases. For instance, during a 10-year analysis of pregnancies with valproate prescriptions in Italian regions such as Tuscany and Emilia-Romagna, there were 172 cases in Emilia-Romagna and 490 cases in Tuscany. In comparison, 353 pregnancies in the UK were exposed to valproate.^14^ The similarities and respective differences towards other countries could be related to both the sociodemographic characteristics of the countries but also may be due to the peculiarities of the pregnancy detection algorithms in different databases. Each region in the aforementioned study^14^ used a different EHR system and a distinct algorithm to identify pregnancy episodes, which could be a limitation.

Regarding usage patterns, when assessing the prevalence of all ASMs globally, there is a clear decrease in use throughout pregnancy by trimester across all the countries assessed. For instance, the use decreases from 5.9‰ in the first trimester of pregnancy to approximately 2‰ in the third trimester, being valproate a 28.6% of those prescriptions.^14^

However, as opposed to other studies, the overall prevalence of valproate prescriptions differs from the results of studies in other settings such as Denmark.^32^ Our prevalence results indicate a significantly higher prevalence compared with studies that focus on claims rather than prescriptions.^33^

Our research also reveals that the measures imposed by the PRAC of the EMA in 2014 had a significant impact.^10^ These measures stipulated that valproate and related substances (valproic acid, valproate and valpromide) should not be used in girls, women of childbearing potential and pregnant women unless alternative treatments are ineffective or not tolerated. Additionally, valproate and related substances should be contraindicated for the prophylaxis of migraine attacks in pregnancy and in women of childbearing potential who are not using effective contraception. These guidelines were updated in 2018^11^ to include a PPP. However, the impact of these latter measures could not be assessed in our study due to the lack of sufficient observational time.

Nevertheless, our study’s data demonstrate the effects of these regulations. There is evidence indicating a shift in the prevalence of valproate prescriptions during pregnancy after 2014, mirroring findings in similar studies that focus on both pregnancy episodes and women of gestational age.^14^,^16^ While some investigations^15^ concentrated on measures instituted in 2018 (with a different methodology), our study, constrained by limited information and time points, chose to evaluate those implemented in 2014. Consistent with trends observed in preceding studies, the use of alternative antiepileptic medications, such as levetiracetam and lamotrigine, witnessed a concurrent rise.^20 32^

Because the current database lacks a mother–child link, it was not feasible to analyse the impact of valproate on offspring during pregnancy, as done in prior studies.^34 35^ Efforts to conduct this analysis will be made in future research. Despite this limitation, we managed to analyse pregnancy outcomes, revealing a predominant occurrence of vaginal deliveries, followed by abortions (spontaneous and induced). This aspect of the research distinguishes it from other cohort database studies.^14^

Concerning health issues (table 1), we should highlight that the most frequent health issue associated with a valproate prescription is epilepsy, followed by anxiety. It is noteworthy, in turn, that 37.08% of pregnancy episodes presented, health issue associated with the year prior to the pregnancy onset, ‘nicontine dependence’. This diagnosis would not only affect the health of mothers during pregnancy but could also have implications for the newborn, which should be closely evaluated.

In comparison to similar research conducted in other countries, the health issues identified closely resemble those observed in other European nations such as France and the UK,^14^ with a notable prevalence of epilepsy and anxiety disorders. Conversely, in the USA, pregnant women exposed to ASM exhibited a higher prevalence of psychiatric disorders, followed by epilepsy and pain diagnoses.^36^ It is also noteworthy that migraine does not appear among the associated comorbidities when compared with other studies,^16^ but this could be because it is not an indication of valproate in Spain. Nevertheless, there is a considerable quantity of missing information on the health issues, and thus, these results must be evaluated with caution.

The aforementioned limitation is common among studies based on data extracted from EHRs, which were originally designed for clinical purposes. As an additional example, within our dataset, certain instances of Voluntary Interruptions of Pregnancy may be erroneously categorised as spontaneous abortions. Abortion in EHRs is not consistently recorded and different models for its register protecting women’s privacy may be difﬁcult. Also, the correct classiﬁcation of abortion in spontaneous, elective or induced, and the outcome registered in SIDIAP did not specify the abortion type, so cases could be spontaneous abortions or induced/elective ones.^37^ Consequently, we encounter challenges in accurately distinguishing between these categories, leading us to tally them collectively.

Moreover, the study’s reliance on an observational design hinders the establishment of causal relationships, emphasising correlations over direct causation. While the study focuses on prescription data for ASM, potential drawbacks exist, such as overlooking dosage and posology. Additionally, the exclusion of billing data introduces the possibility of information gaps. It is important to note that the present study lacks information on pregnancies that are followed up in hospitals (referred from primary care due to a high risk of complications) or in private settings.

At the time of conducting the present study, data on maternal breast feeding were not available, preventing us from determining whether it occurred in pregnancy-exposed episodes resulting in a live-born child. The potential impact of parental valproic acid intake on newborns is currently under evaluation in other studies,^38^ and various regulatory agencies have issued recommendations to healthcare professionals on this matter.^39 40^ Both areas represent intriguing fields for further exploration and investigation.

On the other hand, our study is fortified by a validated methodology, leveraging a validated database^22 23^ and the creation of an algorithm^21^ to ensure the reliability of the data.

It is noteworthy to mention that, globally, there are relatively few studies specifically focused on medication patterns in cohorts of pregnant women. The current study focuses on a cohort of pregnant women in Catalonia and their respective prescriptions for antiepileptic medications. No studies of similar characteristics had been conducted previously in this specific population, and to our knowledge, none have been carried out in Spain. Therefore, this study sheds light on the use of valproate in Spain in pregnant women and allows us to compare our findings with other countries.

Furthermore, considering the impact of the decrease in valproate prescriptions, we can confirm the effectiveness of the strategies employed by local authorities to adhere to RMMs, such as warnings in prescription programmes for medical doctors.^41^ Similar strategies could be implemented in other countries or regions if necessary. While these results may seem promising, the RMMs aimed to prevent any pregnant woman or those of childbearing age from being prescribed valproate. However, the challenging management of the condition, unplanned pregnancies and the lack of information regarding the associated risks with such medication hinder the achievement of this goal.

The findings of the present study align with the results of previous studies. An attempt has been made to profile in-depth the characteristics of pregnancies exposed to valproate, but, however, the use of different algorithms for pregnancy detection in each of the databases of other studies,^14^ as well as the methodology employed, hinder the comparison and generalisation of the results obtained, to the detriment of available scientific evidence. We should aim for greater consensus in the analysis methodology to maximise the pharmacological safety of pregnant patients, who already have a limitation of evidence of drug use for ethical reasons.

Emphasising the critical nature of decision-making in prescribing medications during pregnancy, our study underscores the need for careful consideration and informed choices. The impact of such decisions influences maternal and fetal health outcomes. It highlights the pivotal role healthcare professionals play and emphasises the importance of a thoughtful and evidence-based approach to prescribing.

## supplementary material

10.1136/bmjopen-2024-085167 Supplementary file 1

## Data Availability

Data are available on reasonable request.
