# Supplementary material for: Women’s health in focus: Real-world data on valproate prescriptions during pregnancy – a cohort study in Catalonia (Spain)
Source: BMJ Open. 2024 Aug 12;14(8):e085167. doi: 10.1136/bmjopen-2024-085167 (PMC11337672; doi:10.1136/bmjopen-2024-085167)
Supplement: Supplementary file 1 [file bmjopen-14-8-s001.pdf]

Supplementary Material

Table 1S: ICD10 –Diagnosis

ICD-10 Codes	Health issues
G40.909	Epilepsy, not specified
F41.9	Unspecified anxiety disorder
F17.200	Nicotine dependance: unspecified, uncomplicated
E66.9	Obesity
L29.2	Pruritus
F32.9	Major Depressive Disorder, Single Episode, Unspecified
J40	Chronic obstructive pulmonary disease

Table 2S: Medea Index. Deprivation index (based on five indicators related to work, education, housing conditions) calculated at the census tract level and available for urban areas (MEDEA=1 least deprived; MEDEA=5 Most deprived)

Quintiles	Assigned Status
**U1**	High Socioeconomic Status
**U2**	Moderate Socioeconomic Status
**U3**	Average Socioeconomic Status
**U4**	Low Socioeconomic Status
**U5**	Extreme Socioeconomic Deprivation

TABLE 3S: PREVALENCE OF VPA PRESCRIPTIONS (‰) BY PREGNANCY INTERVAL

Maternal interval	N	D	‰	CI (95%)
3 months before			3.13‰	2.80-3.51‰
First trimester	284	96980	2.92‰	2.60-3.28‰

Second trimester			1.96‰	1.76-2.29‰
Third trimestrer	130	76980	1.69‰	1.42-2.00‰
3 months afer			2.25‰	1.96-2.57‰
6 months after	235	9185	2.55‰	2.25,2.90‰

N (NUMERATOR): EPISODES OF PREGNANCY BY INTERVAL REQUIRING PRESCRIPTION

D (DENOMINATOR): PREGNANCY EPISODES BY INTERVAL

3 MONTHS BEFORE: DATE OF PREGNANCY START - 3 MONTHS

3 MONTHS AFTER: DATE OF PREGNANCY END + 3 MONTHS

6 MONTHS AFTER: DATE OF PREGNANCY END + 6 MONTHS

TABLE 4S: CUMULATIVE INCIDENCE OF VPA PRESCRIPTIONS (‰) BY PREGNANCY INTERVAL

Maternal interval	N	D	‰	IC (95%)
3 months before			0.80	0.63, 1.00
First trimester	60	96880	0.61	0.47, 0.79
Second trimester			0.31	0.20, 0.46
Third trimester	33	76828	0.42	0.29, 0.60
3 months afer			0.83	0.66, 1.00
6 months after	121	91748	1.31	1.09, 1.57

N (NUMERATOR): EPISODES OF PREGNANCY BY INTERVAL REQUIRING PRESCRIPTION (1 YEAR WASHOUT)

D (DENOMINATOR): PREGNANCY EPISODES BY INTERVAL (1 YEAR WASHOUT)

3 MONTHS BEFORE: DATE OF PREGNANCY START - 3 MONTHS

3 MONTHS AFTER: DATE OF PREGNANCY END + 3 MONTHS

6 MONTHS AFTER: DATE OF PREGNANCY END + 6 MONTHS

Tabla 5S: Monthly Prevalence of VPA prescriptions.

Month	Number of prescriptions during pregnancy	Total population	Prevalence	CI 95% (lower)	CI 95% (upper)
**January / 2012**	15	9928	0.0009	0.0009	0.0024
**February 2012**	17	9806	0.0010	0.0010	0.0027
**March / 2012**	18	9730	0.0011	0.0011	0.0029
**April / 2012**	19	9568	0.0014	0.0012	0.0030

**May /2012**	22	9540	0.0013	0.0015	0.0034
**June /2012**	19	9372	0.0020	0.0012	0.0031
**July /2012**	17	9281	0.0018	0.0011	0.0029
**August /2012**	18	9095	0.0019	0.0012	0.0031
**September /2012**	18	8929	0.0020	0.0012	0.0031
**October /2012**	19	8894	0.0021	0.0013	0.0033
**November /2012**	18	8716	0.0020	0.0013	0.0032
**December /2012**	17	8771	0.0019	0.0012	0.0031
**January /2013**	18	8828	0.0020	0.0012	0.0032
**February /2013**	18	8811	0.0020	0.0012	0.0032
**March /2013**	18	8822	0.0020	0.0012	0.0032
**April /2013**	20	8816	0.0022	0.0014	0.0035
**May /2013**	17	8811	0.0019	0.0012	0.0030
**June /2013**	16	8904	0.0017	0.0011	0.0029
**July/2013**	13	8973	0.0014	0.0008	0.0024
**August/2013**	13	8874	0.0014	0.0008	0.0025
**September /2013**	15	8826	0.0016	0.0010	0.0028
**October/2013**	18	8766	0.0020	0.0012	0.0032
**November /2013**	14	8622	0.0016	0.0009	0.0027
**December/2013**	16	8713	0.0018	0.0011	0.0029
**January/2014**	16	8690	0.0018	0.0011	0.0029
**February /2014**	24	8617	0.0027	0.0018	0.0041
**March/2014**	22	8728	0.0025	0.0016	0.0038
**April /2014**	21	8612	0.0024	0.0015	0.0037
**May /2014**	20	8618	0.0023	0.0015	0.0035
**June /2014**	19	8565	0.0022	0.0014	0.0034
**July /2014**	20	8561	0.0023	0.0015	0.0036
**August /2014**	19	8458	0.0022	0.0014	0.0035
**September/2014**	22	8478	0.0025	0.0017	0.0039
**October /2014**	22	8319	0.0026	0.0017	0.0040
**November /2014**	22	8232	0.0026	0.0017	0.0040
**December /2014**	20	8362	0.0023	0.0015	0.0036
**January /2015**	17	8396	0.0020	0.0012	0.0032
**February /2015**	19	8292	0.0022	0.0014	0.0035
**March/2015**	18	8366	0.0021	0.0013	0.0033
**April /2015**	16	8310	0.0019	0.0011	0.0031
**May/2015**	17	8267	0.0020	0.0012	0.0032
**June /2015**	16	8163	0.0019	0.0012	0.0031
**July /2015**	14	8069	0.0017	0.0010	0.0029
**August /2015**	16	8010	0.0019	0.0012	0.0032
**September/2015**	20	8001	0.0024	0.0016	0.0038

**October /2015**	16	7951	0.0020	0.0012	0.0032
**November/2015**	15	7942	0.0018	0.0011	0.0031
**December /2015**	16	7998	0.0020	0.0012	0.0032
**January /2016**	15	7938	0.0018	0.0011	0.0031
**February /2016**	18	7887	0.0022	0.0014	0.0036
**March/2016**	20	7878	0.0025	0.0016	0.0039
**April /2016**	16	7843	0.0020	0.0012	0.0033
**May /2016**	14	7874	0.0017	0.0010	0.0029
**June/2016**	15	7827	0.0019	0.0011	0.0031
**July /2016**	15	7695	0.0019	0.0011	0.0032
**August /2016**	9	7621	0.0011	0.0006	0.0022
**September /2016**	10	7460	0.0013	0.0007	0.0024
**October /2016**	10	7446	0.0013	0.0007	0.0024
**November/2016**	11	7356	0.0014	0.0008	0.0026
**December /2016**	8	7398	0.0010	0.0005	0.0021
**January /2017**	8	7420	0.0010	0.0005	0.0021
**February /2017**	7	7304	0.0009	0.0004	0.0019
**March/2017**	10	7289	0.0013	0.0007	0.0025
**April/2017**	10	7200	0.0013	0.0007	0.0025
**May /2017**	11	7202	0.0015	0.0008	0.0027
**June /2017**	11	7025	0.0015	0.0008	0.0028
**July /2017**	11	6897	0.0015	0.0008	0.0028
**August/2017**	11	6717	0.0016	0.0009	0.0029
**September/2017**	6	6655	0.0009	0.0004	0.0019
**October/2017**	7	6570	0.0010	0.0005	0.0021
**November/2017**	11	6610	0.0016	0.0009	0.0029
**December/2017**	10	6618	0.0015	0.0008	0.0027
**January/2018**	12	6609	0.0018	0.0010	0.0031
**February/2018**	10	6574	0.0015	0.0008	0.0027
**March/2018**	8	6675	0.0011	0.0006	0.0023
**April/2018**	7	6743	0.0010	0.0005	0.0021
**May/2018**	7	6790	0.0010	0.0004	0.0021
**June/2018**	6	6759	0.0008	0.0004	0.0019
**July/2018**	9	6705	0.0013	0.0007	0.0025
**August/2018**	8	6622	0.0012	0.0006	0.0023
**September/2018**	6	6479	0.0009	0.0004	0.0020
**October/2018**	7	6496	0.0010	0.0005	0.0022
**November/2018**	6	6451	0.0009	0.0004	0.0020
**December/2018**	6	6486	0.0009	0.0004	0.0020
**January/2019**	6	6475	0.0009	0.0004	0.0020
**February/2019**	6	6399	0.0009	0.0004	0.0020
**March/2019**	5	6472	0.0007	0.0003	0.0018

**April/2019**	4	6448	0.0006	0.0002	0.0015
**May/2019**	2	6427	0.0003	0.000000008	0.0011
**June/2019**	1	6412	0.0001	0.000000002	0.0008
**July/2019**	3	6346	0.0004	0.0001	0.0013
**August/2019**	3	6225	0.0004	0.0001	0.0014
**November/2019**	5	6129	0.0008	0.0003	0.0019
**October/2019**	6	5975	0.0010	0.0004	0.0021
**November/2019**	5	5815	0.0008	0.0003	0.0020
**December/2019**	5	5616	0.0008	0.0003	0.0020

Table 6S. Monthly prevalence of LMT prescripcions during pregnancy

Month	Number of prescriptions during pregnancy	Total population	Prevalence	CI 95% (lower)	CI 95% (upper)
**January / 2012**	13	9928	0.0013	0.0007	0.0022
**February 2012**	14	9806	0.0014	0.0008	0.0023
**March / 2012**	11	9730	0.0011	0.0006	0.0020
**April / 2012**	11	9568	0.0011	0.0006	0.0020
**May /2012**	11	9540	0.0011	0.0006	0.0020
**June /2012**	10	9372	0.0010	0.0005	0.0019
**July /2012**	11	9281	0.0011	0.0006	0.0021
**August /2012**	11	9095	0.0012	0.0006	0.0021
**September /2012**	10	8929	0.0011	0.0006	0.0020
**October /2012**	13	8894	0.0014	0.0008	0.0024
**November /2012**	15	8716	0.0017	0.0010	0.0028
**December /2012**	17	8771	0.0019	0.0012	0.0031
**January /2013**	19	8828	0.0021	0.0013	0.0033
**February /2013**	18	8811	0.0020	0.0012	0.0032
**March /2013**	17	8822	0.0019	0.0012	0.0030
**April /2013**	20	8816	0.0022	0.0014	0.0035
**May /2013**	20	8811	0.0022	0.0014	0.0035
**June /2013**	18	8904	0.0020	0.0012	0.0031
**July/2013**	21	8973	0.0023	0.0015	0.0035
**August/2013**	20	8874	0.0022	0.0014	0.0034

**September /2013**	16	8826	0.0018	0.0011	0.0029
**October/2013**	16	8766	0.0018	0.0011	0.0029
**November /2013**	18	8622	0.0020	0.0013	0.0032
**December/2013**	17	8713	0.0019	0.0012	0.0031
**January/2014**	15	8690	0.0017	0.0010	0.0028
**February /2014**	14	8617	0.0016	0.0009	0.0027
**March/2014**	16	8728	0.0018	0.0011	0.0029
**April /2014**	16	8612	0.0018	0.0011	0.0030
**May /2014**	18	8618	0.0020	0.0013	0.0032
**June /2014**	19	8565	0.0022	0.0014	0.0034
**July /2014**	20	8561	0.0023	0.0015	0.0036
**August /2014**	15	8458	0.0017	0.0010	0.0029
**September/2014**	18	8478	0.0021	0.0013	0.0033
**October /2014**	18	8319	0.0021	0.0013	0.0034
**November /2014**	17	8232	0.0020	0.0012	0.003
**December /2014**	16	8362	0.0019	0.0011	0.0031
**January /2015**	15	8396	0.0017	0.0010	0.0029
**February /2015**	15	8292	0.0018	0.0010	0.0029
**March/2015**	14	8366	0.0016	0.0009	0.0028
**April /2015**	16	8310	0.0019	0.0011	0.0031
**May/2015**	12	8267	0.0014	0.0008	0.0025
**June /2015**	12	8163	0.0014	0.0008	0.0025
**July /2015**	14	8069	0.0017	0.0010	0.0029
**August /2015**	15	8010	0.0018	0.0011	0.0030
**September/2015**	21	8001	0.0026	0.0017	0.0040
**October /2015**	16	7951	0.0020	0.0012	0.0032
**November/2015**	16	7942	0.0020	0.0012	0.0032
**December /2015**	14	7998	0.0017	0.0010	0.0029
**January /2016**	16	7938	0.0020	0.0012	0.00327
**February /2016**	18	7887	0.0022	0.0014	0.0036
**March/2016**	17	7878	0.0021	0.0013	0.0034

**April /2016**	18	7843	0.0022	0.0014	0.0036
**May /2016**	20	7874	0.0025	0.0016	0.0039
**June/2016**	22	7827	0.0028	0.0018	0.0042
**July /2016**	21	7695	0.0027	0.0017	0.0041
**August /2016**	20	7621	0.0026	0.0016	0.0040
**September /2016**	23	7460	0.0030	0.0020	0.0046
**October /2016**	22	7446	0.0029	0.0019	0.0044
**November/2016**	23	7356	0.0031	0.0020	0.0046
**December /2016**	21	7398	0.0028	0.0018	0.0043
**January /2017**	20	7420	0.0026	0.0017	0.0041
**February /2017**	19	7304	0.0026	0.0016	0.0040
**March/2017**	18	7289	0.0024	0.0015	0.0039
**April/2017**	15	7200	0.0020	0.0012	0.0034
**May /2017**	14	7202	0.0019	0.0011	0.0032
**June /2017**	16	7025	0.0022	0.0014	0.0036
**July /2017**	19	6897	0.0027	0.0017	0.0042
**August/2017**	16	6717	0.0023	0.0014	0.0038
**September/2017**	17	6655	0.0025	0.0015	0.0040
**October/2017**	21	6570	0.0031	0.0020	0.0048
**November/2017**	21	6610	0.0031	0.0020	0.0048
**December/2017**	19	6618	0.0028	0.0018	0.0044
**January/2018**	20	6609	0.0030	0.0019	0.0046
**February/2018**	18	6574	0.0027	0.0017	0.0043
**March/2018**	19	6675	0.0028	0.0018	0.0044
**April/2018**	22	6743	0.0032	0.0021	0.0049
**May/2018**	21	6790	0.0030	0.0020	0.0047
**June/2018**	17	6759	0.0025	0.0015	0.0040
**July/2018**	21	6705	0.0031	0.0020	0.0047
**August/2018**	23	6622	0.0034	0.0023	0.0052
**September/2018**	27	6479	0.0041	0.0028	0.0060
**October/2018**	29	6496	0.0044	0.0031	0.0064
**November/2018**	29	6451	0.0044	0.0031	0.0064

**December/2018**	28	6486	0.0043	0.0029	0.0062
**January/2019**	26	6475	0.0040	0.0027	0.0058
**February/2019**	22	6399	0.0034	0.0022	0.0052
**March/2019**	21	6472	0.0032	0.0021	0.0049
**April/2019**	21	6448	0.0032	0.0021	0.0049
**May/2019**	20	6427	0.0031	0.0020	0.0048
**June/2019**	20	6412	0.0031	0.0020	0.0048
**July/2019**	19	6346	0.0029	0.0019	0.0046
**August/2019**	18	6225	0.0028	0.0018	0.0045
**November/2019**	16	6142	0.0026	0.0016	0.0042
**October/2019**	17	6016	0.0028	0.0017	0.0045
**November/2019**	19	5878	0.0032	0.0020	0.0050
**December/2019**	19	5709	0.0033	0.0021	0.0051

Table 7S. Monthly prevalence of LVTprescripcions during pregnancy

Month	Number of prescriptions during pregnancy	Total population	Prevalence	CI 95% (lower)	CI 95% (upper)
**January / 2012**	9	9928	0.0009	0.0004	0.0017
**February 2012**	10	9806	0.0010	0.0005	0.0018
**March / 2012**	11	9730	0.0011	0.0006	0.0020
**April / 2012**	14	9568	0.0014	0.0008	0.0024
**May /2012**	13	9540	0.0013	0.0007	0.0023
**June /2012**	14	9372	0.0014	0.0008	0.0025
**July /2012**	14	9281	0.0015	0.0008	0.0025
**August /2012**	12	9095	0.0013	0.0007	0.0023
**September /2012**	13	8929	0.0014	0.0008	0.0024
**October /2012**	13	8894	0.0014	0.0008	0.0024
**November /2012**	13	8716	0.0014	0.0008	0.0025

**December /2012**	13	8771	0.0014	0.0008	0.0025
**January /2013**	13	8828	0.0014	0.0008	0.0025
**February /2013**	17	8811	0.0019	0.0012	0.0030
**Marvch /2013**	18	8822	0.0020	0.0012	0.0032
**April /2013**	19	8816	0.0021	0.0013	0.0033
**May /2013**	22	8811	0.0024	0.0016	0.0037
**June /2013**	27	8904	0.0030	0.0020	0.0044
**July/2013**	24	8973	0.0026	0.0017	0.0039
**August/2013**	23	8874	0.0025	0.0017	0.0038
**September /2013**	23	8826	0.0026	0.0017	0.0039
**October/2013**	23	8766	0.0026	0.0017	0.0039
**November /2013**	20	8622	0.0023	0.0015	0.0035
**December/2013**	17	8713	0.0019	0.0012	0.0031
**January/2014**	20	8690	0.0023	0.0014	0.0035
**February /2014**	17	8617	0.0019	0.0012	0.0031
**March/2014**	19	8728	0.0021	0.0013	0.0033
**April /2014**	20	8612	0.0023	0.0015	0.0035
**May /2014**	21	8618	0.0024	0.0015	0.0037
**June /2014**	20	8565	0.0023	0.0015	0.0036
**July /2014**	22	8561	0.0025	0.0016	0.0038
**August /2014**	22	8458	0.0026	0.0017	0.0039
**September/2014**	21	8478	0.0024	0.0016	0.0037
**October /2014**	21	8319	0.0025	0.0016	0.0038
**November /2014**	23	8232	0.0027	0.0018	0.0041
**December /2014**	23	8362	0.0027	0.0018	0.0041
**January /2015**	26	8396	0.0030	0.0021	0.0045
**February /2015**	24	8292	0.0028	0.0019	0.0043
**March/2015**	23	8366	0.0027	0.0018	0.0041
**April /2015**	21	8310	0.0025	0.0016	0.0038
**May/2015**	21	8267	0.0025	0.0016	0.0038
**June /2015**	20	8163	0.0024	0.0015	0.0037

**July /2015**	23	8069	0.0028	0.0019	0.0042
**August /2015**	23	8010	0.0028	0.0019	0.0043
**September/2015**	24	8001	0.0029	0.0020	0.0044
**October /2015**	25	7951	0.0031	0.0021	0.0046
**November/2015**	21	7942	0.0026	0.0017	0.0040
**December /2015**	20	7998	0.0025	0.0016	0.0038
**January /2016**	21	7938	0.0026	0.0017	0.0040
**February /2016**	27	7887	0.0034	0.0023	0.0049
**March/2016**	30	7878	0.0038	0.0026	0.0054
**April /2016**	26	7843	0.0033	0.0022	0.0048
**May /2016**	25	7874	0.0031	0.0021	0.0046
**June/2016**	23	7827	0.0029	0.0019	0.0044
**July /2016**	28	7695	0.0036	0.0025	0.0052
**August /2016**	23	7621	0.0030	0.0020	0.0045
**September /2016**	24	7460	0.0032	0.0021	0.0047
**October /2016**	24	7446	0.0032	0.0021	0.0047
**November/2016**	30	7356	0.0040	0.0028	0.0058
**December /2016**	29	7398	0.0039	0.0027	0.0056
**January /2017**	34	7420	0.0045	0.0032	0.0063
**February /2017**	35	7304	0.0047	0.0034	0.0066
**March/2017**	35	7289	0.0048	0.0034	0.0066
**April/2017**	30	7200	0.0041	0.0029	0.0059
**May /2017**	30	7202	0.0041	0.0029	0.0059
**June /2017**	31	7025	0.0044	0.0031	0.0062
**July /2017**	30	6897	0.0043	0.0030	0.0062
**August/2017**	28	6717	0.0041	0.0028	0.0060
**September/2017**	23	6655	0.0034	0.0023	0.0051
**October/2017**	27	6570	0.0041	0.0028	0.0059
**November/2017**	27	6610	0.0041	0.0028	0.0059
**December/2017**	24	6618	0.0036	0.0024	0.0053
**January/2018**	24	6609	0.0036	0.0024	0.0053

**February/2018**	25	6574	0.0038	0.0025	0.0056
**March/2018**	28	6675	0.0041	0.0029	0.0060
**April/2018**	30	6743	0.0044	0.0031	0.0063
**May/2018**	26	6790	0.0038	0.0026	0.0056
**June/2018**	29	6759	0.0042	0.0029	0.0061
**July/2018**	32	6705	0.0047	0.0033	0.0067
**August/2018**	32	6622	0.0048	0.0034	0.0068
**September/2018**	31	6479	0.0047	0.0033	0.0067
**October/2018**	30	6496	0.0046	0.0032	0.0065
**November/2018**	31	6451	0.0048	0.0033	0.0068
**December/2018**	31	6486	0.0047	0.0033	0.0067
**January/2019**	28	6475	0.0043	0.0029	0.0062
**February/2019**	26	6399	0.0040	0.0027	0.0059
**March/2019**	31	6472	0.0047	0.0033	0.0067
**April/2019**	30	6448	0.0046	0.0032	0.0066
**May/2019**	29	6427	0.0045	0.0031	0.0064
**June/2019**	27	6412	0.0042	0.0028	0.0061
**July/2019**	27	6346	0.0042	0.0029	0.0061
**August/2019**	28	6225	0.0044	0.0031	0.0064
**November/2019**	30	6142	0.0048	0.0034	0.0069
**October/2019**	27	6016	0.0044	0.0030	0.0065
**November/2019**	28	5878	0.0047	0.0032	0.0068
**December/2019**	25	5709	0.0043	0.0029	0.00645

Table 8S. ATC CODES.

ATC CODE	DRUG	Abbreviation
N03AG01	Valproate	VPA
N03AX09	Lamotrigine	LMT
N03AX14A	Levetiracetam	LVT
