# Supplementary material for: Women’s health in focus: Real-world data on valproate prescriptions during pregnancy – a cohort study in Catalonia (Spain)
Source: BMJ Open. 2024 Aug 12;14(8):e085167. doi: 10.1136/bmjopen-2024-085167 (PMC11337672; doi:10.1136/bmjopen-2024-085167)
Supplement: Supplementary file 2 [file bmjopen-2024-085167.reviewer_comments.pdf]

PEER REVIEW HISTORY

BMJ Open publishes all reviews undertaken for accepted manuscripts. Reviewers are asked to complete a checklist review form (**http://bmjopen.bmj.com/site/about/resources/checklist.pdf**) and are provided with free text boxes to elaborate on their assessment. These free text comments are reproduced below.

ARTICLE DETAILS

**TITLE (PROVISIONAL)**	Women's health in focus. Real-World Data on Valproate Prescriptions During Pregnancy: A Cohort Study in Catalonia
**AUTHORS**	Bellas, Lucía; Camacho-Arteaga, Lina; Giner-Soriano, Maria; Prats-Uribe, Albert; Aguilera, Cristina; Vedia Urgell, Cristina; Agustí, Antonia

VERSION 1 – REVIEW

**REVIEWER**	Simões-Wüst, Ana Paula<br>University Hospital Zurich
**REVIEW RETURNED**	25-Mar-2024

**GENERAL COMMENTS**	The study by Bellas et al. addresses an important topic in the treatment of pregnant women, namely the use of valproate during pregnancy. For their study, they extracted data from Catalonia primary care electronic health records. Data on valproate exposures were available for 302 pregnancies (276 women). I found particularly interesting that the prescriptions data are shown by trimester and that the periods before and after pregnancy were as well studied. I would like to recommend its publication if following aspects would be improved or clarified.<br><br>- In the abstract, page 3, lines 14-16, the authors mention that the data lack "information on pregnancies that are followed up in hospitals (referred from Primary Care due to a high risk of complications) or in private settings". Since epilepsy is associated with a high risk of complications during pregnancy, this is an important limitation of the present work. Therefore, it is important to add to the title and to the objective (page 4, lines 13-16), that the work was done with data from primary care and to duly discuss this aspect in the manuscript.<br>- Page 7, lines 35-43: Could the authors illustrate their statements with numbers? For instance instead of writing that the results align with previous ones, to mention the prevalence data so that the reader can agree or not whether they really align? The discussion is not long, so I think you can develop more these ideas.<br>- Page 7, lines 46-53: Also here, the authors could be more precise and mention for instance which regulatory agencies exactly and which measures were instituted.<br>- Page 8, lines 6-11: It is peculiar that 37% of the women on valproate had an alcohol withdrawal syndrome. Could the authors check and describe how "alcohol withdrawal syndrome" has been defined?<br>- Page 8, lines 45-48: 302 pregnancies is not a lot, so the authors should delete the "substantial number of participants"<br>- Page 4, lines 43-44: Is it meant that only full pregnancy episodes were considered? If yes, "only" should be added.

	- I was a bit irritated to read that epilepsy is a co-morbidity (being valproate known as an epilepsy-medication). Would it is better to speak about “other indications than pregnancy”?<br>- Page 6, lines 50-51: What is it meant exactly with washout?

**REVIEWER**	ter Horst, P.G.J.<br>Isala Hospitals, Clinical Pharmacy
**REVIEW RETURNED**	31-May-2024

**GENERAL COMMENTS**	dear Authors, thanks for your manuscript. I have amajor problem with this manuscript, as what does this study add to the scientific community? there are several reports about incidence/prevalence of valproate prescribing during pregnancy. So, why is your study important? what is the attributal value of it with reference to other reports? different findings?, methods much better?<br>A minor issue concerns a point made in the discussion: most frequent comorbidity associated to valproate prescription is epilepsy. what is the main morbidity?

VERSION 1 – AUTHOR RESPONSE

Reviewer 1- Dr. Ana Paula Simões-Wüst, University Hospital Zurich

Comments to the Author: I would like to recommend its publication if the following aspects could be improved or clarified.

- In the abstract, page 3, lines 14-16, the authors mention that the data lack “information on pregnancies that are followed up in hospitals (referred from Primary Care due to a high risk of complications) or in private settings”. Since epilepsy is associated with a high risk of complications during pregnancy, this is an important limitation of the present work. Therefore, it is important to add to the title and to the objective (page 4, lines 13-16), that the work was done with data from primary care and to duly discuss this aspect in the manuscript.

The information that might be missing regarding pregnancies followed in hospitals mainly and exclusively pertains to clinical parameters of pregnancy monitoring, days of hospitalization, and the clinical characteristics of newborns. The repository used for data analysis does record outpatient prescriptions from both hospitals and primary care, while diagnoses/health issues are those registered by the primary care physician. Since valproate is an outpatient prescription (even if prescribed by a hospital physician, it is uploaded to ECAP and thus recorded in our system), it does not pose a limitation in identifying pregnancies exposed to valproate. Corresponding clarifications have been added in the abstract (page 3, lines 14-16), Strengths and limitations section and Design and Methods section.

- Page 7, lines 35-43: Could the authors illustrate their statements with numbers? For instance instead of writing that the results align with previous ones, to mention the prevalence data so that the reader can agree or not whether they really align? The discussion is not long, so I think you can develop more these ideas.
Added.

Overall prevalence, prevalence by trimesters and prescription patterns, align with findings from research carried in other countries (14,20), especially those corresponding to Italian databases. For

instance, during a 10-year analysis of pregnancies with valproate prescriptions in Italian regions such as Tuscany and Emilia-Romagna, there were 172 cases in Emilia-Romagna and 490 cases in Tuscany. In comparison, 353 pregnancies in the UK were exposed to valproate (14).The similarities and respective differences towards other countries could be related to both the sociodemographic characteristics of the countries but also may be due to the peculiarities of the pregnancy detection algorithms in different databases. Each region in the aforementioned study (14) used a different electronic health record system and a distinct algorithm to identify pregnancy episodes, which could be a limitation.

Regarding usage patterns, when assessing the prevalence of all antiseizure medications globally, there is a clear decrease in use throughout pregnancy by trimester across all the countries assessed. For instance, the use decreases from 5.9 ‰ in the first trimester of pregnancy to approximately 2 ‰ in the third trimester, being valproate a 28.6% of those prescriptions (14).

- Page 7, lines 46-53: Also here, the authors could be more precise and mention for instance which regulatory agencies exactly and which measures were instituted:

Our research also reveals that the measures imposed by the Pharmacovigilance Risk Assessment Committee (PRAC) of the European Medicines Agency in 2014 had a significant impact (10). These measures stipulated that valproate and related substances (valproic acid, valproate, and valpromide) should not be used in girls, women of childbearing potential, and pregnant women unless alternative treatments are ineffective or not tolerated. Additionally, valproate and related substances should be contraindicated for the prophylaxis of migraine attacks in pregnancy and in women of childbearing potential who are not using effective contraception. These guidelines were updated in 2018 (11) to include a pregnancy prevention program. However, the impact of these latter measures could not be assessed in our study due to the lack of sufficient observational time.

- Page 8, lines 6-11: It is peculiar that 37% of the women on valproate had an alcohol withdrawal syndrome. Could the authors check and describe how “alcohol withdrawal syndrome” has been defined?
We have identified an error in the coding of the current data. That 37% did not correspond to alcohol withdrawal syndrome, but to nicotine dependence. It has been corrected in the manuscript and supplementary figures. The "comorbidities" (from now on "health issues") refer to health problems recorded by the primary care physician in the medical history, and we do not have linked diagnoses with prescriptions, so we do not know if all prescriptions match the detected comorbidity. We will assess this limitation in the discussion section

- Page 8, lines 45-48: 302 pregnancies are not a lot, so the authors should delete the “substantial number of participants”: *Modified. Also from the Strengths and Limitations section.*

- Page 4, lines 43-44: Is it meant that only full pregnancy episodes were considered? If yes, “only” should be added: *Added*

- I was a bit irritated to read that epilepsy is a co-morbidity (being valproate known as an epilepsy-medication). Would it is better to speak about “other indications than pregnancy”?
“Comorbidities” are health issues recorded by the Primary Care Physician in each visit. In our case, prescriptions are not linked to specific comorbidities, so we assessed them all globally, not being able to tell exactly what was the prescriber intended indication, so we were trying to avoid that term. We also consider that the term comorbidity might be confusing, so we will be substituting it by “health issues”. We also clarified this issue in the manuscript (Design and Methods section).

- Page 6, lines 50-51: What is it meant exactly with washout?

"Washout" refers to the period of time necessary without a prescription to consider a case as new in calculations of cumulative incidence. In our study, patients who had an active valproate prescription during the previous year could not be considered as incident cases. We will include a sentence in the manuscript to clarify this.

Reviewer: 2
Dr. P.G.J. ter Horst, Isala Hospitals

dear Authors, thanks for your manuscript. I have a major problem with this manuscript, as what does this study add to the scientific community? there are several reports about incidence/prevalence of valproate prescribing during pregnancy. So, why is your study important? what is the attributal value of it with reference to other reports? different findings?, methods much better?

Dear reviewer,

Thank you for your response. Below is our reply, incorporating references from the manuscript.

While there are indeed some reports on the use of valproate during pregnancy (14, 20), most of them focus on prescriptions of antiepileptic drugs in general during pregnancy, rather than specifically on valproate and without considering the periods before and after pregnancy. Moreover, these publications were issued shortly after the implementation of EMA measures (10), allowing little time to assess their real impact. Other studies do not specifically target pregnant women but rather women of childbearing potential in general (15, 16).

Valproate is a teratogenic drug that poses health risks to offspring (1), and recent recommendations have been made regarding its use by parents (40). Therefore, its periconceptional use must be regularly evaluated from a population and pharmacovigilance standpoint by regulatory agencies, alongside the use of available therapeutic alternatives. This need is underscored by recent EMA requests for population-based studies using standardized methodologies to enable cross-country comparisons (16) and requests for studies on the impact of valproic acid intake by parents (40). The outcomes of these studies are likely to prompt regulatory measures at the European level.

Efforts are increasingly being made to conduct such studies in a standardized manner to enhance comparability between countries and tailor measures accordingly. Moreover, to our knowledge, no study of this nature has been conducted in Catalonia or Spain before.

Our study relies on an algorithm for detecting pregnancies in electronic health records (21), recently published, which could serve as a guide in future periconceptional studies, promoting data harmonization and comparison across countries. Prevalence and incidence studies were conducted using tools employed in Common Data Models (28, 29), facilitating generalization of calculations and data for potential use in other studies.

These methodological innovations have enabled us to assess the real impact of EMA measures (the main focus of our study) in a region where such measures had not previously been studied, proposing diverse methodologies for their evaluation in other countries as well. Importantly, our evaluation extends beyond the prevalence of valproate prescriptions to include the usage of alternative medications. We also propose strategies which could be implemented in other countries or regions if necessary (42). Gathering evidence on population-level medication use enriches scientific knowledge and informs policy decisions aimed at enhancing the safety of those who take them, a fundamental principle of pharmacovigilance.

A minor issue concerns a point made in the discussion: most frequent comorbidity associated to valproate prescription is epilepsy. what is the main morbidity?
"Comorbidities" are health issues recorded by the Primary Care Physician in each visit. In our case, prescriptions are not linked to specific comorbidities, so we assessed them all globally. We also consider that the term comorbidity might be confusing, so we will be substituting it by "health issues". We also clarified this issue in the manuscript (Design and Methods section).

VERSION 2 – REVIEW

**REVIEWER**	ter Horst, P.G.J.<br>Isala Hospitals, Clinical Pharmacy
**REVIEW RETURNED**	10-Jul-2024

**GENERAL COMMENTS**	thanks for the calrification, now, together with the adequatly adressed comments , acceptable for publication
